# Tuberculous constrictive pericarditis diagnosed by cardiac magnetic resonance imaging: A case report

**DOI:** 10.1097/MD.0000000000048394

**Published:** 2026-04-24

**Authors:** Gang Zhou, Li Yang, Ling Wu, Kaichuang Deng, Yan Long

**Affiliations:** a Department of Radiology, Gejiu People’s Hospital, Gejiu City, Yunnan Province, China.

**Keywords:** cardiac magnetic resonance imaging, constrictive pericarditis, pharmacologic treatment, pleural effusion, tuberculous pericarditis

## Abstract

**Rationale:**

Constrictive pericarditis (CP) is a diagnostic challenge due to its nonspecific and insidious presentation.

**Patient concerns:**

A 38-year-old man presented with bilateral lower limb edema and exertional dyspnea.

**Diagnoses:**

Cardiac magnetic resonance imaging demonstrated pericardial thickening, late gadolinium enhancement, and interventricular septal bounce. Tuberculous CP was considered based on imaging findings, positive interferon-gamma release assay, therapeutic response, and epidemiologic background.

**Interventions:**

The patient was treated with anti-tuberculosis therapy without surgical pericardiectomy.

**Outcomes:**

Follow-up imaging showed near-complete resolution of pericardial thickening and effusion, with sustained clinical improvement.

**Lessons:**

This case highlights the value of cardiac magnetic resonance imaging in the early diagnosis of inflammatory CP and supports the role of medical therapy in selected patients.

## 1. Introduction

Constrictive pericarditis (CP) is a clinical syndrome characterized by a rigid, often thickened or calcified pericardium that restricts diastolic filling, resulting in elevated venous pressures and reduced cardiac output. Although idiopathic or viral pericarditis remains the predominant cause in developed countries, tuberculosis (TB) continues to be a leading etiology of CP worldwide, accounting for 31.65 cases per 1000 person-years.^[1]^ The pathophysiological hallmarks of CP include dissociation between intrathoracic and intracardiac pressures, ventricular interdependence, and impaired diastolic filling.^[2]^ Clinical diagnosis is often delayed due to nonspecific symptoms such as peripheral edema, dyspnea, or ascites, as well as the absence of overt signs of pericardial disease during the early stages.^[3]^ Diagnostic imaging – particularly echocardiography and computed tomography (CT) – may suggest pericardial thickening or effusion; however, these findings are often nonspecific or inconclusive.

Cardiac magnetic resonance (CMR) imaging is increasingly recognized as a valuable modality for the evaluation of pericardial diseases, providing detailed assessment of cardiac anatomy, function, adjacent extracardiac structures, pericardial thickening, effusion, and pericardial fluid characteristics. Growing evidence indicates that pericardial enhancement on CMR not only facilitates the diagnosis of pericarditis but also offers prognostic insight into the likelihood of reversible pericardial inflammation, thereby guiding therapeutic decision-making in selected patients.^[4]^

This case is significant for several reasons. First, it illustrates an atypical presentation of tuberculous CP characterized predominantly by pleural involvement and minimal systemic symptoms, thereby complicating early diagnosis. Second, the diagnosis was supported noninvasively through CMR imaging, which demonstrated pericardial thickening and delayed gadolinium enhancement – representing key features of active pericardial inflammation. Cine imaging further revealed interventricular septal bounce, a hallmark of constrictive physiology. Notably, unlike many cases that necessitate surgical pericardiectomy, this patient achieved complete clinical and radiologic resolution following pharmacologic anti-TB therapy. By presenting this case, we underscore the critical role of CMR in the early identification of CP, which enables timely and targeted medical management.

## 2. Case presentation

A 38-year-old male patient was admitted to our hospital on May 8, 2024, with a chief complaint of bilateral lower limb edema lasting for 2 weeks. The edema developed insidiously without an identifiable precipitating factor, was symmetrically distributed, and exhibited diurnal variation – milder in the morning and more pronounced in the evening. The patient also reported chest tightness and exertional dyspnea, both of which improved with rest. He had self-administered diuretics, but the symptoms showed minimal improvement. He denied any history of chronic conditions such as hypertension, diabetes mellitus, or coronary artery disease, nor did he report any previous infectious diseases.

**Figure 1.**
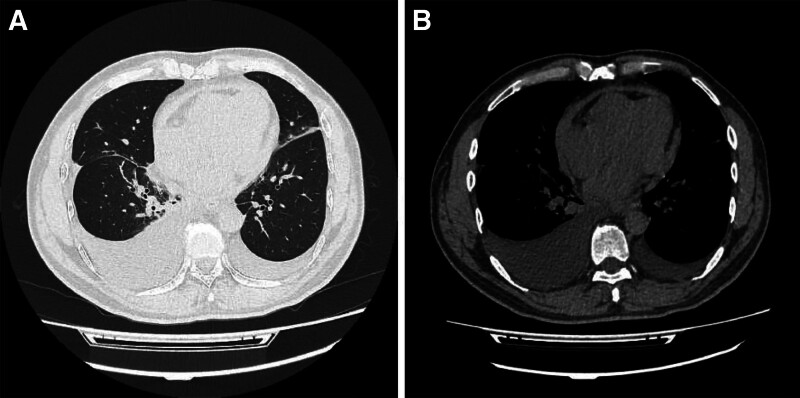
Pretreatment chest computed tomography (CT) imaging findings. (A) Axial lung window CT image shows bilateral pleural effusions, more prominent on the right side, with diffuse peribronchial thickening and patchy opacities in both lungs, indicative of inflammatory changes. (B) Axial mediastinal window CT image demonstrates significant pericardial thickening without evidence of pericardial calcification.

Percussion demonstrated dullness below the 8th rib on the right and below the 9th rib on the left, and breath sounds were diminished bilaterally without adventitious sounds. Prominent pitting edema was present in both lower limbs. The initial diagnosis was edema of unclear etiology accompanied by bilateral pleural effusions. Laboratory findings revealed an elevated B-type natriuretic peptide level of 165.00 pg/mL, while cardiac enzymes remained within normal limits. Blood tests showed mild monocytosis (9.5%), a hematocrit of 36.0%, and a red cell distribution width (RDW-CV) of 15.4%, with no elevation in C-reactive protein. Electrocardiography demonstrated T-wave inversion in leads V3–V6. Chest CT (Fig. 1) revealed bilateral pleural effusions – more prominent on the right side – along with lower lobe infiltrates, partial right lower lobe atelectasis, and pericardial thickening suggestive of inflammatory changes. Echocardiography indicated a small pericardial effusion, impaired left ventricular diastolic function, and preserved systolic function. However, classic echocardiographic features of CP, such as interventricular septal bounce or respiratory variation in transvalvular flow velocities, were not documented, and specific quantitative parameters according to the Mayo Clinic criteria were not assessed. On May 9, 2024, the patient underwent right-sided thoracentesis and drainage, which yielded 600 mL of orange-tinged, mildly turbid fluid. Biochemical analysis confirmed transudative characteristics of the pleural effusion, and TB DNA testing was negative. Based on Light criteria and the B-type natriuretic peptide level, the pleural effusion was determined to be related to cardiac dysfunction.

**Figure 2.**
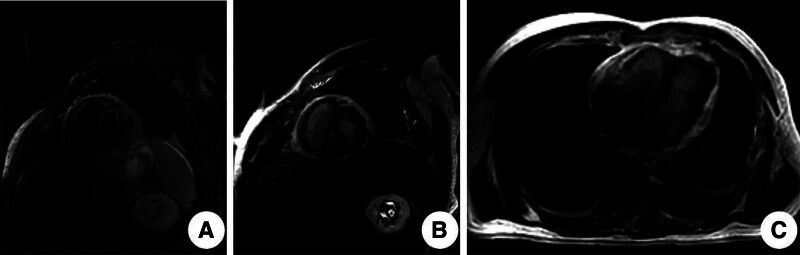
Pretreatment cardiac magnetic resonance (CMR) imaging. (A) T2-weighted short-axis image shows pericardial thickening, with the thickest area measuring approximately 1.2 cm. (B, C) Late gadolinium enhancement (LGE) sequences in short-axis and 4-chamber views demonstrate pericardial enhancement, consistent with active pericarditis.

CMR (Fig. 2) confirmed pericardial thickening, with a maximal pericardial thickness of approximately 1.2 cm on T2-weighted short-axis images. Late gadolinium enhancement (LGE) sequences in both short-axis and 4-chamber views demonstrated pericardial enhancement, consistent with active pericarditis. Cine imaging revealed interventricular septal bounce and mild impairment of left ventricular wall motion (Supplementary Video 1). These findings favored an inflammatory, potentially reversible form of CP rather than chronic fibrotic disease. Based on the clinical presentation and cardiac MR findings, the patient was diagnosed with CP, accompanied by a small pericardial effusion and bilateral pleural effusions. The patient subsequently requested referral to a tertiary cardiovascular center for further management.

**Figure 3.**
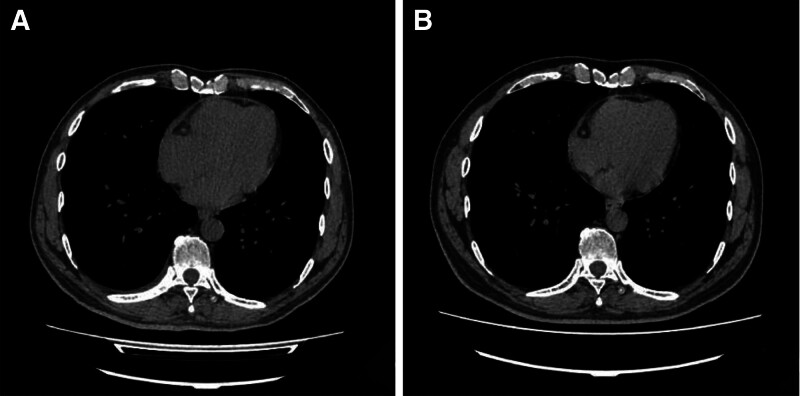
Follow-up chest computed tomography (CT) after treatment. (A) Axial mediastinal window CT image at 3 months after treatment shows a marked reduction in pericardial thickening compared to baseline. (B) Axial mediastinal window CT image at 9 months shows near-complete resolution of pericardial thickening, with no signs of calcification or effusion.

The patient was evaluated at Fuwai Cardiovascular Hospital of Yunnan Province, where an interferon-gamma release assay for TB was positive, and tuberculous CP was considered the most likely diagnosis. Anti-TB therapy was initiated on May 24, 2024, consisting of isoniazid, rifampin, pyrazinamide, and ethambutol. After nearly 3 months of 4-drug therapy, treatment was transitioned to isoniazid and rifampin maintenance therapy, which has been continued to date in accordance with standard TB treatment principles. Follow-up chest CT performed on September 2, 2024 (Fig. 3A), demonstrated reduced pericardial thickening and effusion, resolution of bilateral pulmonary infiltrates and left pleural effusion, and only a small residual right-sided pleural effusion. A subsequent CT on February 25, 2025 (Fig. 3B), revealed further improvement, with near-complete resolution of pericardial thickening and effusion.

At the most recent outpatient follow-up on November 24, 2025, the patient remained functionally stable, with no recurrence of lower limb edema, chest tightness, or exertional dyspnea. Both clinical symptoms and imaging abnormalities improved progressively without the need for surgical intervention.

## 3. Discussion

CP is a rare but serious condition with diverse etiologies and nonspecific clinical manifestations, often leading to diagnostic delays. In TB-endemic regions, tuberculous pericarditis remains a leading cause of CP; however, its presentation is frequently insidious, with symptoms such as peripheral edema and pleural effusion often misattributed to other systemic or cardiac conditions.^[5]^ This case exemplifies such a diagnostic challenge: the patient presented primarily with bilateral lower extremity edema and pleural effusions, but did not exhibit classical signs of advanced heart failure or pericardial involvement. Echocardiography revealed a small pericardial effusion with impaired left ventricular diastolic function, and chest CT demonstrated pericardial thickening without calcification. CMR, however, provided crucial diagnostic insights, demonstrating pericardial thickening, LGE, and interventricular septal bounce – findings indicative of both active inflammation and constrictive physiology. This case highlights the value of CMR not only in morphological assessment but also in functional evaluation, enabling timely recognition of CP prior to the development of irreversible pericardial fibrosis. Given the potentially reversible nature of inflammation-driven CP, particularly in tuberculous etiologies, early diagnosis supports medical management and may eliminate the need for surgical pericardiectomy.^[6]^

Despite advances in diagnostic imaging and understanding of pericardial disease, CP continues to be one of the most challenging cardiac conditions to diagnose. In the context of tuberculous pericarditis, which accounts for a significant proportion of CP cases in TB-endemic regions, early-stage presentation often mimics heart failure or pulmonary pathology, which often leads to misdiagnosis and delayed treatment. Classic diagnostic modalities such as transthoracic echocardiography and CT provide useful structural information but are limited in detecting pericardial inflammation or early-stage hemodynamic compromise. CMR has gained recognition as a powerful tool due to its ability to visualize pericardial thickness, inflammation, and dynamic physiology – such as septal bounce and ventricular coupling. LGE can differentiate active inflammatory constriction from chronic fibrotic pericarditis, which is critical for therapeutic decision-making.^[7,8]^ However, in practice, the diagnosis is often complicated by overlapping clinical signs, variable sensitivity of imaging modalities, and the absence of microbiologic confirmation of TB in pericardial fluid or tissue. Although the interferon-gamma release assay result was positive, it does not confirm active TB infection in isolation. However, in this case, the combination of characteristic imaging findings, a favorable therapeutic response to anti-TB therapy, and the epidemiologic background supported the diagnosis of tuberculous CP. CMR imaging provides detailed anatomic and functional assessment; however, it cannot directly identify the causative pathogen in pericardial diseases. Alternative etiologies, including post-surgical, idiopathic, or autoimmune pericarditis, remain possible in the absence of histologic confirmation. This case reflects such diagnostic uncertainty: pleural effusion with subtle cardiac symptoms initially suggested alternative etiologies, and CMR played a decisive role in supporting the diagnosis of active CP. These findings underscore the importance of multimodality imaging and clinical suspicion, especially in regions with a high prevalence of TB.

This case strengthens the existing understanding that early inflammatory CP may be medically reversible if diagnosed in a timely manner. In this patient, full clinical and radiological resolution was achieved without surgical intervention, consistent with previous findings that early medical therapy can lead to the reversal of pericardial constriction.^[9]^ Among the tools available for assessing this complex hemodynamic condition and guiding treatment, few are as important as CMR, especially for its capacity to assess pericardial inflammation. This capability is crucial for identifying patients with potentially reversible disease and for preventing unnecessary pericardiectomy in the presence of active inflammation.^[10]^ Similar to previous reports where multimodality imaging altered diagnostic and therapeutic pathways – such as idiopathic ascending aortitis and combined myocardial infarction with pulmonary embolism – this case reinforces that advanced imaging is pivotal not only in diagnosis but also in guiding management decisions and preventing unnecessary invasive procedures.^[11,12]^

## 4. Conclusion

This case illustrates the vital importance of considering CP in patients presenting with unexplained pleural effusions and subtle cardiac symptoms, particularly in TB-endemic areas. It underscores the diagnostic value of CMR in detecting pericardial inflammation and constrictive physiology, enabling timely and targeted therapy. The successful clinical and radiological resolution with anti-TB treatment supports the potential reversibility of active inflammatory CP. This case emphasizes that, in selected patients with stable hemodynamics and imaging features of active inflammation, conservative medical management may be a feasible and effective alternative to surgical intervention. Early identification and targeted treatment are essential for improving outcomes and reducing long-term complications.

## Author contributions

**Conceptualization:** Gang Zhou, Ling Wu.

**Data curation:** Gang Zhou, Li Yang.

**Formal analysis:** Gang Zhou.

**Investigation:** Gang Zhou, Li Yang, Kaichuang Deng, Yan Long.

**Methodology:** Gang Zhou, Yan Long.

**Project administration:** Gang Zhou, Ling Wu, Yan Long.

**Software:** Gang Zhou, Kaichuang Deng.

**Supervision:** Gang Zhou, Li Yang, Ling Wu, Kaichuang Deng.

**Validation:** Gang Zhou.

**Visualization:** Gang Zhou, Ling Wu, Kaichuang Deng, Yan Long.

**Writing – original draft:** Gang Zhou.

**Writing – review & editing:** Ling Wu.
